# Evaluating the effectiveness of a multi-faceted inpatient diabetes management program among hospitalised patients with diabetes mellitus

**DOI:** 10.1186/s40842-020-00107-2

**Published:** 2020-11-05

**Authors:** Shih Ling Kao, Ying Chen, Yilin Ning, Maudrene Tan, Mark Salloway, Eric Yin Hao Khoo, E Shyong Tai, Chuen Seng Tan

**Affiliations:** 1 Department of Medicine, National University Hospital and National University Health System, Singapore, Singapore; 2 Department of Medicine, Yong Loo Lin School of Medicine, National University of Singapore and National University Health System, Singapore, Singapore; 3 Saw Swee Hock School of Public Health, National University of Singapore and National University Health System, Singapore, Singapore; 4 NUS Graduate School for Integrative Sciences and Engineering, National University of Singapore, Singapore, Singapore; 5 Department of Surgery, Yong Loo Lin School of Medicine, National University of Singapore and National University Health System, Singapore, Singapore

**Keywords:** Diabetes mellitus, Healthcare quality improvement, Inpatient hypoglycemia, Inpatient hyperglycemia

## Abstract

**Background:**

Diabetes mellitus (DM) is one of the most common chronic diseases. Individuals with DM are more likely to be hospitalised and stay longer than those without DM. Inpatient hypoglycemia and hyperglycemia, which are associated with adverse outcomes, are common, but can be prevented through hospital quality improvement programs.

**Methods:**

We designed a multi-faceted intervention program with the aim of reducing inpatient hypoglycemia and hyperglycemia. This was implemented over seven phases between September 2013 to January 2016, and covered all the non-critical care wards in a tertiary hospital. The program represented a pragmatic approach that leveraged on existing resources and infrastructure within the hospital. We calculated glucometric outcomes in June to August 2016 and compared them with those in June to August 2013 to assess the overall effectiveness of the program. We used regression models with generalised estimating equations to adjust for potential confounders and account for correlations of repeated outcomes within patients and admissions.

**Results:**

We observed significant reductions in patient-days affected by hypoglycemia (any glucose reading < 4 mmol/L: OR = 0.71, 95% CI: 0.61 to 0.83, *p* <  0.001), and hyperglycemia (any glucose reading > 14 mmol/L: OR = 0.84, 95% CI: 0.71 to 0.99, *p* = 0.041). Similar findings were observed for admission-level hypoglycemia and hyperglycemia. Further analyses suggested that these reductions started to occur four to 6 months post-implementation.

**Conclusions:**

Our program was associated with sustained improvements in clinically relevant outcomes. Our described intervention could be feasibly implemented by other secondary and tertiary care hospitals by leveraging on existing infrastructure and work force.

**Supplementary information:**

**Supplementary information** accompanies this paper at 10.1186/s40842-020-00107-2.

## Background

Diabetes mellitus (DM) is one of the most common chronic diseases worldwide and is of public health significance, particularly in Singapore where the prevalence of DM is high at 11% [1]. Individuals with DM are more likely to be hospitalised, and have longer hospitalisations than those without DM [2]. The estimated prevalence of inpatient diabetes can be as high as 20 to 40%, especially among the elderly hospitalised population [3, 4].

Glycemic control in hospitalised patients with DM is often suboptimal with occurrence of both hypoglycemia and hyperglycemia [5, 6]. These are associated with adverse hospitalisation outcomes such as increased mortality and duration of hospitalisation [7–10]. Intercurrent illness can destabilise glycemic control. Iatrogenic factors like under-treatment, over-treatment, and medication errors also contribute significantly to inpatient hypoglycemia and hyperglycemia. These iatrogenic causes are preventable. It is now recognised that system-wide approaches are required to address the safety of DM patients in hospital. Prevention of hypoglycemia and hyperglycemia has been adopted as quality measures for inpatient care [2, 11, 12].

Published strategies to improve inpatient DM management vary [13–20] from designing inpatient glucose monitoring policies coupled with education programs, to more targeted approaches involving deployment of glycemic control teams. The former is relatively easy to implement, but the long-term effectiveness is uncertain. The latter is labour-intensive and may be impractical for a large tertiary hospital with high inpatient DM prevalence. Some hospitals have also leveraged on the use of electronic glucose management systems to improve glycemic control, which involve investments in technology infrastructure [21] It is likely that no single approach is superior, but a combination of strategies may be required to optimise inpatient DM management. However, large hospitals aiming to improve inpatient DM management may face resource constraints, and other competing patient safety priorities.

We describe here a real-world implementation of a hospital-wide inpatient DM management program in the non-critical care wards of a large tertiary hospital. The objective was to reduce inpatient hypoglycemia and hyperglycemia. We utilised a pragmatic and multi-component approach, leveraging on existing infrastructure and work force. We then examined if the intervention was associated with reduction in hypoglycemia and hyperglycemia rates, and when the intervention started to take effect.

## Methods

### Intervention

The program was implemented in a 1200-bed tertiary hospital in Singapore, which manages 64,000 admissions per year. The program consisted of 7 components. The components of the program were:

1. A multidisciplinary inpatient diabetes safety committee was established – This committee consisted of major stakeholders involved in the delivery of inpatient DM care, including endocrinologists, diabetes specialist nurses, pharmacists, nurses and hospital administrators.
2. Establishment of inpatient diabetes guidelines – Inpatient diabetes guidelines encompassing the common scenarios in inpatient diabetes care were developed to standardise care. These include management of hyperglycemia, hypoglycemia, nil by mouth (i.e., fasting) orders, hyperglycemic crises (including diabetes ketoacidosis and hyperglycemic hyperosmolar state), and transition to home. These guidelines were co-developed by the inpatient diabetes safety committee together with residents, pharmacists, and nurses to create a sense of ownership. Efforts were taken to simplify management algorithms without compromising on safety, to ensure that the guidelines were practicable by the healthcare workers responsible for delivering the care. For example, the guidelines describe how to use the patient’s usual home regimen with a supplemental insulin scale. If glycemic control remains suboptimal, guidance was provided on initiating basal-bolus insulin treatment in hospital. They were made easily accessible on the hospital intranet page for quick reference, with link outs from the electronic health record.
3. Enhancements of existing hospital electronic medical system – The hospital utilised an electronic prescribing system. Prior to the program, there was widespread use of insulin sliding scales as monotherapy, and prescribers used a variety of scales or created their own. To facilitate prescribing according to the inpatient DM guidelines, and to reduce variation in care, standardised electronic supplemental insulin order sets were created. We also designed an alert system that alerted doctors to patients with hypoglycemia using existing rule engines available within the hospital information technology (IT) system.
4. Education of residents, nurses, and pharmacists – Ward residents, nurses and pharmacists were educated on the inpatient DM management guidelines. This was facilitated on a large scale using an online interactive case-based e-learning course [22]. We also embedded inpatient DM management education modules into the staff orientation programs.
5. Active identification and intervention for inpatients with hypoglycemia and hyperglycemia by ward pharmacists – Point-of-care (POC) blood glucose (BG) results within our hospital were electronically captured and transmitted to a central laboratory data repository. Analytics were developed to identify patients with hypoglycemia, near-hypoglycemia, and hyperglycemia on a daily basis. We had an existing workflow in which ward pharmacists would regularly review inpatient medications of hospitalised patients. We leveraged on and enhanced this existing review process by providing ward pharmacists with a worklist of patients with suboptimal glycemic control. They will then review and discuss treatment changes with the primary ward physician. Challenging cases would be referred to the inpatient DM consulting service lead by an endocrinologist.
6. Improvements to nursing documentation – We improved existing nursing documentation in the hospital to facilitate adherence to the inpatient DM guidelines. Prior to the program, inpatient POC BG readings were measured and charted by nurses on a paper chart. An improved glucose monitoring chart was developed together with ward nurses. Colour codes were used to highlight alert levels for clinical action. A documentation template for hypoglycemia rescue was developed to facilitate the appropriate rescue of inpatient hypoglycemia by nurses.
7. Identification of ward-level nursing champions – Ward-level nursing champions were identified and trained to be resource persons within their wards. They conducted regular audits of inpatient DM nursing management within their wards. Specifically, adherence to mild hypoglycemia management and nil by mouth management were audited, and each ward was encouraged to develop ground-up initiatives to improve areas of deficiencies. They also met regularly to share their audit findings and best practices.

The program was implemented in all adult non-critical care wards which included medical, surgical and psychiatry wards, but excluded pediatric and labour wards. Components 1 to 3 of the program were implemented at the start of the program across all non-critical care wards. The remaining components 4 to 7 were implemented as a package progressively in phases between September 2013 to January 2016. Each phase consisted of a cluster of three to six non-critical care wards. We completed implementation in each cluster before moving on to the next phase. There were a total of seven phases.

### Assessment of effectiveness of the program

#### Time period of comparisons

We performed two comparisons to assess the effectiveness of the program in improving glucometric outcomes. In the first comparison, we compared glucometric outcomes during June to August 2013 (3 months before the start of the program) with June to August 2016 (6 months after the last phase of implementation). This comparison aimed to assess if the entire program achieved reductions in hypoglycemia and hyperglycemia across all non-critical care adult wards in the hospital.

In the second comparison, we assessed the short-term change in glucometrics for each of the seven phases of implementation from September 2013 to January 2016 to see when the intervention started to have an effect. We compared the glucometrics across three time periods within each phase: 1) Pre-implementation (3 months pre-implementation), 2) Post-implementation period 1 (first to third months post-implementation), and 3) Post-implementation period 2 (fourth to sixth months post-implementation). This comparison assessed the immediate effectiveness post-implementation. We classified admissions that spanned across time periods based on the admission date. For example, if a patient was admitted in pre-implementation period but discharged in post-implementation period 1, this admission of the patient would be classified as a pre-implementation admission.

#### Study population

**Fig. 1:**
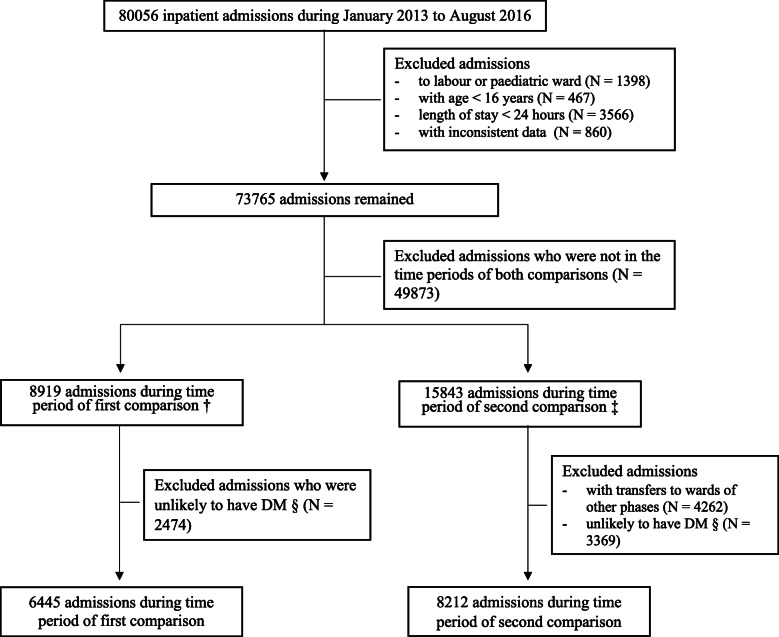
Flow diagram of admissions included in the study. †The first comparison compared June to August 2013 against June to August 2016. ‡The second comparison compared three time periods for each phase: 1) Pre-implementation (3 months pre-implementation), 2) Post-implementation period 1 (first to third months post-implementation), and 3) Post-implementation period 2 (fourth to sixth months post-implementation for each phase). §An admission was considered as likely on DM if the patient had 1) any prescription of DM medications, or 2) any capillary blood glucose reading > 11.1 mmol/L, during the inpatient admission

There were 42,618 patients consisting of 80,056 admissions to non-critical care wards (637,588 patient-days) identified during the specified time periods (Fig. 1). We excluded admissions shorter than 24 h, as the program is unlikely to have an effect on the glycemic control of very short hospitalisations. We also excluded patients with age less than 16 years, and admissions to pediatric and labor wards, as the inpatient DM guidelines do not apply to these populations. We excluded 860 (1.1%) admissions due to data inconsistencies across the study periods, including inconsistent age, gender and ethnicity information across repeated admissions, and chronically inconsistent admission, discharge or laboratory test timings (Fig. 1). For the second comparison, we also excluded admissions in which the patient was transferred to wards outside of the existing phase of implementation.

We excluded admissions that were unlikely to have DM. To identify admissions which were likely to have DM, we applied the following criteria: 1) prescription of DM medications, or 2) any capillary BG reading > 11.1 mmol/L [23] during the admission. As HbA1c was highly correlated with capillary BG readings, we found that adding HbA1c > 6.5% to our selection criteria did not significantly increase the number of admissions identified. Details of the population inclusion flow is presented in Fig. 1.

### Glucometrics

‘Glucometrics’, is a terminology for glucose performance metrics, obtained from standardised methods of analysing glucose data. Glucometrics can be expressed using the following denominators or levels: individual glucose tests, per patient-day, per admission [24]. We utilised patient-day and admission level glucometrics in our analysis, as these are more clinically relevant [25].

Glucose readings performed in the emergency department and intensive care units were excluded from analysis. All remaining POC capillary BG measurements performed during these admissions were used for analysis where wards that admitted predominantly medical (e.g., general medicine), surgical (e.g., hepatobiliary surgery) and non-medical and non-surgical (e.g., obstetrics and gynaecology) patients were classified as medical, surgical and others respectively. We further classified admissions into medical and surgical wards if the patient stayed in a medical and surgical ward throughout the stay respectively, and “others” if the patient was transferred across different types of wards during the stay, or if the admission was to a non-medical and non-surgical ward.

For hypoglycemia, we measured the rates of patient-days (or admissions) with any episode of hypoglycemia defined at cut-offs of 4, 3 or 2.5 mmol/L [26, 27], and rates of admissions with recurrent hypoglycemia (two or more days with any BG <  4 mmol/L). For hyperglycemia, we measured the rates of patient-days (or admissions) with any episode of hyperglycemia at cut-offs of 14 or 20 mmol/L [15, 16, 28]. We used the hyperglycemia index (HGI) for measuring exposure to significant hyperglycemia, calculated by taking the area under the interpolated curve of all glucose values within the admission above 14 mmol/L divided by the length of stay (LOS) [29]. We chose 14 mmol/L as a threshold for moderately severe hyperglycemia. We also measured the odds of mean glucose reading in the desired range of 4 to 10 mmol/L, mean glucose, and standard deviation (SD) of glucose values [30, 31]. We also assessed if the intervention was associated with any change in LOS.

### Statistical analysis

The primary outcomes in this study are the glycemic profiles measured by the glucometrics mentioned above. Means with SD were reported for continuous and count outcomes, while counts with percentages were reported for binary outcomes for each time periods. To visualize the trend of glycemic control with hypoglycemia and hyperglycemia, we used statistical control p-chart [32–34] with the control limits set at ± 3SD. We log-transformed mean and SD of glucose, and HGI to reduce their skewness, and accounted for potential confounders and correlations within patients and admissions by using linear regression models with generalised estimating equations (GEE) [35] when estimating the intervention effect by having intervention period as a categorical dependent variable. Similar to continuous outcomes, we used the Poisson and logistic regression models with GEE to model counts (i.e., LOS in days) and binary outcomes (i.e., patient-day or admission having any episode of hypoglycemia, hyperglycemia, and patient-day or admission with the mean glucose reading in desired range). The estimated coefficient of the intervention period variable indicated the mean ratio (or odds ratio) between non-intervention and intervention periods for continuous and count outcomes. We specified the working correlation structure as first-order autoregressive and used the ‘sandwich’ estimator to obtain robust standard errors. From the literature we identified a list of variables associated with glycemia profile and accounted for potential confounding by adjusting the intervention effects for demographic variables (i.e., age, gender, ethnicity), admission type (i.e., emergency versus non-emergency admission), type of ward the patients were admitted to (i.e., medical or surgical wards), and likelihood of illness severity (i.e., presence of any one of the following laboratory tests during an admission: albumin, creatinine, C-reactive protein, white blood cells, and troponin I). For LOS, we also adjusted for glycemic profile of patients as a poorer glycemic profile is associated with longer LOS. For the analysis of the intervention effect for the second comparison, we also adjusted for the implementing phase. Among outcomes where the intervention program had significant effect (i.e., *p* <  0.05), we performed additional follow-up analysis to compare the effectiveness of the program between medical and surgical wards and assess whether type of ward was an effect modifier.

We reported corresponding 95% confidence intervals (CIs) with 2-sided *p*-values. We used R version 3.11.1 [36] and its package Generalised Estimating Equation Package (GEEPACK) to perform the analysis [37].

## Results

### Comparison 1: June to august 2013 VS June to august 2016

**Fig. 2:**
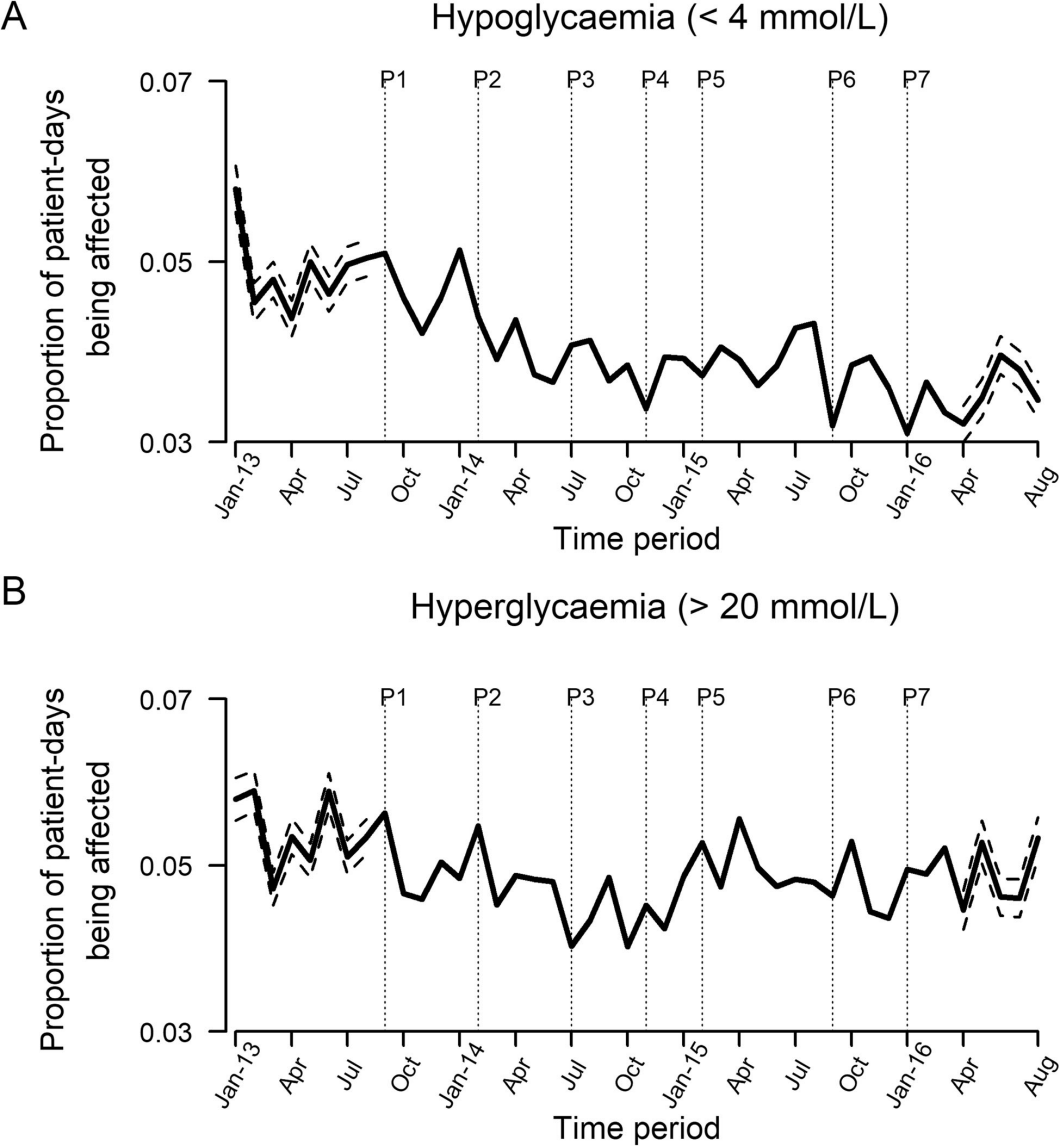
p-chart of hypoglycemia (< 4 mmol/L) and hyperglycemia (> 20 mmol/L). P1 denotes the start of the first phase where components 1–3 of the program was implemented across all non-critical care wards, and components 4–7 in the first phase wards. P2 to P7 denote the implementation of components of 4–7 in phase 2 to 7 respectively. Dotted lines were control limits and were drawn for periods before the start of the program (June to August 2013) and 3 months after the last phase of implementation (June to August 2016)

There were 6445 admissions used in the comparison between June to August 2013 and June to August 2016 (Fig. 1). Table 1 describes the admission profiles. The mean age was 66 to 68 years with a slight male preponderance. The majority of the admissions were to medical wards. There were slightly more admissions through the emergency department, but less severe admissions in 2016 compared to the same time period in 2013 (Table 1). Figure 2 displays the statistical control p-chart for the proportion of patient-days with hypoglycemia (< 4 mmol/L) and hyperglycemia (> 20 mmol/L) over the entire study period where the decline was more pronounced for hypoglycemia than hyperglycemia between June to August 2013 and June to August 2016.

**Table 1:** Profiles of admissions during June to August 2013 compared against June to August 2016

	June to August 2013	June to August 2016	***P***-value^a^
**Admissions**	3315	3130	
**DEMOGRAPHIC**			
**Age (in years), mean (SD)**	67.6 (13.7)	66.4 (13.5)	0.01
**Male, n (%)**	1785 (53.9)	1678 (53.6)	0.861
**Ethnicity, n (%)**			
Chinese	1878 (56.8)	1840 (58.8)	0.359
Indian	477 (14.4)	425 (13.6)	
Malay	675 (20.4)	600 (19.2)	
Others	285 (8.6)	265 (8.5)	
**ADMISSION CHARACTERISTICS**			
**Admissions to, n (%)**			
Medical wards	2776 (83.7)	2549 (81.4)	0.029
Surgical wards	425 (12.8)	473 (15.1)	
Others^b^	114 (3.4)	108 (3.45)	
**Admission via emergency, n (%)**	2238 (67.5)	2214 (70.7)	0.005
**With more severe illness, n (%)**^**c**^	2999 (90.5)	2633 (84.1)	< 0.001
**INFORMATION USED TO IDENTIFY ADMISSIONS WITH DM**			
**DM medications was prescribed, n (%)**	2931 (88.4)	2851 (91.1)	< 0.001
**Any capillary blood glucose reading > 11.1 mmol/L, n (%)**	2849 (85.9)	2557 (81.7)	< 0.001
**Any HbA1c > 6.5% or 48 mmol/L, n (%)**	869 (26.2)	531 (17.0)	< 0.001

^a^For age, Student’s t-test was performed to obtain the *p*-value. For the rest of the variables, Fisher’s exact test was performed to obtain the *p*-values

^b^Others include patients who were transferred across types of wards during admission, or admitted to a non-medical and non-surgical ward

^c^An admission was considered as being more severe if the patient had any of the following laboratory tests performed during the hospital stay, albumin, creatinine, C - reactive protein, white blood cells, and troponin I

Table 2 summarises the key glucometrics for June to August 2013 compared against June to August 2016. There was a reduction in hypoglycemia (< 4 mmol/L) after program implementation. We observed a 29% (95% CI 17 to 39%) reduction in the odds for a patient-day, and a 28% (95% CI 17 to 38%) reduction in the odds of an admission to be affected by hypoglycemia. Improvement was observed irrespective of the severity of hypoglycemia, with more pronounced improvement for more severe hypoglycemia. We observed reductions in recurrent hypoglycemia, with a 35% (95% CI 17 to 48%) reduction in admissions with two or more days affected by hypoglycemia. We did not find any statistically significant interactions between type of ward (i.e., medical vs surgical) and reductions in hypoglycemia at both patient-day and admission levels.

**Table 2:** Comparison of key glucometrics between June to August 2013 and June to August 2016

	June to August 2013	June to August 2016	Effect measures^**a**^ (95% CI)	***P***-value
**Patient-days**	21,399	17,876		
**Admissions**	3315	3130		
**HYPOGLYCEMIA METRICS**				
**Patient-day level**				
**Hypoglycemia, any glucose, n (%)**				
< 4 mmol/L	997 (4.7)	588 (3.3)	0.71 (0.61 to 0.83)	< 0.001
< 3 mmol/L	196 (0.9)	83 (0.5)	0.51 (0.38 to 0.7)	< 0.001
< 2.5 mmol/L^b^	55 (0.3)	22 (0.1)	0.52 (0.28 to 0.96)	0.035
**Admission level**				
**Hypoglycemia, any glucose, n (%)**				
< 4 mmol/L	556 (16.8)	384 (12.3)	0.72 (0.62 to 0.83)	< 0.001
< 3 mmol/L	141 (4.3)	71 (2.3)	0.54 (0.4 to 0.73)	< 0.001
< 2.5 mmol/L^b^	42 (1.3)	17 (0.5)	0.45 (0.26 to 0.78)	0.005
**Recurrence of hypoglycemia, number of days with any glucose < 4 mmol/L, n (%)**				
Exactly 1 day^c^	345 (10.4)	252 (8.1)	0.76 (0.64 to 0.9)	0.002
2 or more days^c^	211 (6.4)	132 (4.2)	0.65 (0.52 to 0.83)	< 0.001
**HYPERGLYCEMIA AND OTHER METRICS**				
**Patient-day level**				
**Hyperglycemia, any glucose, n (%)**				
> 14 mmol/L	5929 (27.7)	4678 (26.2)	0.84 (0.71 to 0.99)	0.041
> 20 mmol/L	1255 (5.9)	843 (4.7)	0.81 (0.66 to 0.99)	0.039
**Mean glucose in desired range, n (%)**				
Within 4–10 mmol/L	13,206 (61.7)	11,357 (63.5)	1.11 (0.93 to 1.33)	0.237
**Mean glucose, mean (SD)**	9.7 (3.3)	9.6 (3.2)	0.98 (0.94 to 1.02)	0.342
**SD of glucose, mean (SD)**	2.4 (1.7)	2.3 (1.6)	0.95 (0.92 to 0.98)	0.001
**Admission level**				
**Hyperglycemia, any glucose, n (%)**				
> 14 mmol/L	1838 (55.4)	1616 (51.6)	0.87 (0.79 to 0.97)	0.01
> 20 mmol/L	608 (18.3)	459 (14.7)	0.79 (0.69 to 0.92)	0.002
**Mean glucose in desired range, n (%)**				
Within 4–10 mmol/L	1986 (59.9)	1957 (62.5)	1.14 (1.02 to 1.27)	0.022
**HGI, mean (SD)**	0.49 (0.99)	0.42 (0.92)	0.68 (0.53 to 0.88)	0.004
**Mean glucose, mean (SD)**	9.8 (2.7)	9.6 (2.6)	0.98 (0.96 to 0.99)	< 0.001
**SD of glucose, mean (SD)**	2.9 (1.4)	2.7 (1.3)	0.95 (0.92 to 0.97)	< 0.001
**LOS (in days), mean (SD)**^**d**^	7.5 (7.9)	7.2 (6.8)	1.01 (0.96 to 1.05)	0.793

^a^The associations between glucometrics and the time periods were fully adjusted with respect to age, gender, ethnicity groups, emergency admission, type of ward, and illness severity status. For mean glucose, SD (standard deviation) of glucose, HGI (Hyperglycemia Index) with threshold being 14 mmol/L, and LOS (length of stay), Poisson regression model with Generalized Estimation Equations (GEE) was applied and mean ratios were reported with 95% CI within the brackets. For other metrics, logistic regression model with GEE was applied and odds ratios were reported with 95% CI within the brackets

^b^Blood glucose < 2.5 mmol/L is considered biochemically severe hypoglycemia

^c^For recurrence of hypoglycemia, the comparison reference group is always admissions without any glucose < 4 mmol/L, i.e., zero days with any glucose < 4 mmol/L.

^d^The associations between length of stay and the time periods were fully adjusted with respect to age, gender, ethnicity groups, emergency admission, type of ward, illness severity status, and glucometrics

For hyperglycemia, there was a 16% (95% CI 1 to 29%) reduction in the odds for a patient-day and a 13% (95% CI 3 to 21%) reduction in the odds for an admission to be affected by hyperglycemia (> 14 mmol/L). The beneficial effect was more pronounced for more severe hyperglycemia. Overall, there was a 14% (95% CI 2 to 27%) increase in odds for an admission to have a mean glucose in the desired range. We also noted a marginal reduction in admission-level mean glucose value, and both patient-day level and admission-level glucose variability. We also observed a 32% (95% CI 12 to 47%) reduction in HGI. We noted that the program was more effective in reducing patient-day hyperglycemia (> 14 mmol/L) among medical wards compared to surgical wards (interaction *p* = 0.002), with a significant 21% (95% CI 5 to 35%) reduction in odds of patient-day hyperglycemia among admissions to medical wards but a significant 41% (95% CI 1 to 97%) increase among admissions to surgical wards (see Table 3). We did not find any statistically significant interactions between type of ward and reductions in mean glucose and HGI at both patient-day and admission levels. We did not observe a statistically significant increase in LOS after program implementation. As less patients were admitted and the proportion prescribed with DM medication was significantly higher between June and August 2016, we performed additional sensitivity analyses that accounted for these factors and similar results were observed.

**Table 3:** Effect modification analysis between June to August 2013 and June to August 2016

	June to August 2013 *N* = 3201^a^	June to August 2016 *N* = 3022^a^	Effect measures^b^ (95% CI)	*P*-value
Patient-days	20,277	17,183		
Patient-days with any glucose > 14 mmol/L, n (%)				
Medical ward	5155 (25.4)	4023 (23.4)	0.79 (0.65 to 0.95)	0.012
Surgical ward	508 (2.5)	535 (3.1)	1.41 (1.01 to 1.97)	0.046

^a^Number of admissions to medical and surgical wards was reported for each time period and it constituted about 97% of total admissions for both time periods

^b^The associations between glucometrics and the time periods were fully adjusted with respect to the implementing phase, age, gender, ethnicity groups, emergency admission, type of ward, and illness severity status, using logistic regression with Generalized Estimation Equations

### Comparison 2: assessment of glucometric changes during the implementation phase

**Fig. 3:**
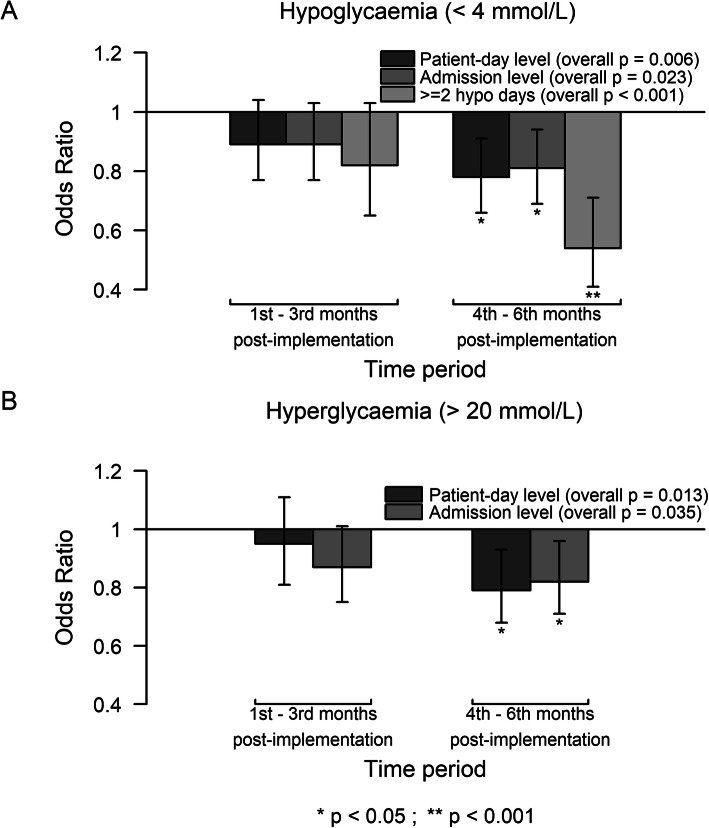
Improvement in hypoglycemia (< 4 mmol/L) and hyperglycemia (> 20 mmol/L) in second comparison. Effect sizes with the 95% confidence interval (vertical line) on hypoglycemia (< 4 mmol/L) and hyperglycemia (> 20 mmol/L) where these outcomes within the first 3 months and subsequent 3 months of post-implementation at each of the 7 phases were compared with those from the 3 months period prior implementation

To determine when the program started to exhibit an effect, we assessed the changes in glucometrics during the implementation process, by comparing across three time periods for each phase (3 months pre-implementation, first to third months post-implementation, and fourth to sixth months post-implementation). The admission profiles were similar among the three periods, apart from a decreasing trend in proportions of emergency and severe admissions (see Table S1 in Additional file 1). We observed reductions in both patient-day and admission level hypoglycemia (< 4 mmol/L) rates, recurrent hypoglycemia rates, and both patient-day and admission level severe hyperglycemia rates (> 20 mmol/L). Overall, the improvements were more pronounced in the fourth to sixth months post-implementation, compared to the first to third months post-implementation (Fig. 3). There was no evidence that the type of ward modified the improvement in these glucometrics. We did not observe significant changes in mean glucose, SD of glucose values, and odds of mean glucose in the desired range. The changes in all patient-day and admission level of these glucometrics across the three time periods are presented in Table S2 in Additional file 1.

## Discussion

We described the implementation of a hospital-wide inpatient diabetes program in a large tertiary hospital. We were able to implement it without large investments in infrastructure or workforce, by leveraging on manpower, workflows and IT systems already in existence. One key characteristic of our program was its multidisciplinary approach. Prior to this, our primary strategy was resident education. However, the high turnover rates due to residency rotations limited its effectiveness. To circumvent this, we engaged ward nurses, pharmacists, and residents through co-development of guidelines, new workflows, and education. We created online education modules to efficiently reach a larger audience of healthcare workers, and reduced time spent on face-to-face teaching. Users were also encouraged to refer regularly to the inpatient diabetes guidelines that were available on the hospital intranet page. Information on how often and which groups referred to these guidelines would have been useful but was unfortunately unavailable, as access was allowed without user identification or password to facilitate ease of use.

We observed a sustained reduction in clinically relevant outcomes, including hypoglycemia rates, recurrent hypoglycemia rates, hyperglycemia rates, and glucose variability. Munoz et al. [15] described a glucose management program which was associated with a sustained reduction of 18.8% in incidence of patient-days with hypoglycemia (< 3.9 mmol/L). Their program was implemented over 3 years, during which specific components of the program were implemented one at a time. Although such a strategy would allow the evaluation of incremental effectiveness with each component, we chose to implement a bundle of changes in groups of wards at a time. This approach allowed the inpatient diabetes safety committee to engage closely with the staff in each cluster of wards undergoing the change. We were also able to make improvements after each phase of implementation with real-time feedback.

van Noord et al. [14] reported a 37% reduction in unadjusted odds of admissions developing hyperglycemia (> 15 mmol/L) after implementing a protocol to standardise inpatient care among 360 patients with DM in a surgery department. The program involved deployment of a DM team consisting of an internist and diabetes specialist nurse to provide consultation at admission, a standardised insulin adjustment scheme, and a glucose monitoring protocol. The reported reduction in hyperglycemia was higher than the reduction of unadjusted odds for admission level hyperglycemia (> 14 and 20 mmol/L: 14 and 23% respectively) observed in our study. This is likely due to targeted interventions by a specialised team. However, van Noord’s approach will be resource-intensive in a large tertiary hospital with a high inpatient prevalence of DM. Comparatively, we were able to demonstrate an improvement in hyperglycemia in a much larger population compared to their study.

Our program was more effective in reducing hypoglycemia compared to hyperglycemia. This was because our glycemic management policies placed a greater emphasis on hypoglycemia prevention, and less on achieving very tight glycemic control in non-critical care wards, where robust evidence that such degrees of control improves outcomes was lacking. We were cognisant that a policy that overemphasises hypoglycemia prevention, may lead to increase in hyperglycemia. Therefore, it was important to develop holistic policies which deal with both hypoglycemia and hyperglycemia. Although we achieved reduction in hyperglycemia while reducing hypoglycemia, this was mainly driven by a decrease in hyperglycemia in medical wards. We noted an increase in rates of patient-day hyperglycemia (> 14 mmol/L) in surgical wards. On the other hand, the program was equally effective in reducing hypoglycemia in both medical and surgical wards. The underlying reason for increased moderate hyperglycemia in surgical wards is not known. One possibility is a greater prevalence of stress hyperglycemia in the post-intervention period. Our hypothesis is that surgical teams may find medication titration to address hyperglycemia more challenging. Potential future strategies to address this include guided algorithms, clinical decision support, or targeted deployment of glycemic control teams in these wards.

We observed only a marginal decrease in mean glucose. Munoz et al. [15] reported a significant decrease of 7.8 mg/dL (about 0.43 mmol/L) in average patient-day mean glucose for each admission. This reduction is greater compared to our study, where we only observed a 0.2 mmol/L reduction in admission level mean glucose for all patients with DM. In another recent study on Asian healthcare, Swee et al. [19] reported a decrease of 1.2 mmol/L in both patient-day and admission level mean glucose after the deployment of a multidisciplinary team to review patients and provide recommendations for inpatient glucose management. The difference might be due to the patient population in the various studies. In Munoz et al’s analysis, they selected patients with hyperglycemia only. In Swee et al’s study, they focused on a specific group of patients who were greater than 21 years old and with at least three glucose measurements being abnormal (< 4 mmol/L or > 10 mmol/L) during a 24-h period. Another reason for the marginal improvement in our study may be the significant reductions in both hypoglycemia and hyperglycemia rates. This is supported by the improvement in glucose variability in our study, which is another indicator that both hypoglycemia and hyperglycemia were reduced. This finding may be highlighted as a limitation of using mean glucose as an outcome measure in such studies. Apart from mean glucose, other glucometrics such as HGI and glucose within range can be measured to reflect changes in hyperglycemia.

There is often a concern that an increased awareness of glucose management by primary teams might result in prolongation of admission to optimize glycemic control. However, we did not observe any clinically significantly increased LOS after the program implementation. The analysis of the change in glucometrics across time was instructive. Both hypoglycemia and hyperglycemia rates took time to improve, with the impact at fourth to sixth months being greater than first to third months post-implementation. This suggests that future analysis of similar intervention programs should consider a time period for implemented changes to take effect, before analysing the full effectiveness of the interventions.

The main strength of this current study is that it is a step-wise implementation in a large tertiary hospital as per a real-world scenario. There are however several limitations to our analysis. This is an observational study and susceptible to confounding. For example, patients were younger and less likely to be with more severe illness between June and August 2016, suggesting the patients in the latter period were less sick and less likely to develop hypoglycemia or hyperglycemia, especially as comorbidities were not collected. As such, we have used regression models to account for these factors or their surrogates when possible. We were not fully able to adjust for disease severity due to a lack of complete data on comorbidities. We used the presence of certain laboratory tests as a surrogate where the underlying assumption was admissions that ordered these tests were likely to be of a more severe nature. We acknowledge that this is not the usual practice of defining disease severity. We had not used the numerical values of these tests to adjust for severity as this would have resulted in the exclusion of about 15% of the study population due to absence of data. We also did not have data on types and doses of steroid use, which is a known contributor of inpatient hyperglycemia.

Our inclusion criteria may have included some individuals with stress hyperglycemia without established diabetes. However, we did not exclude this group of patients from receiving interventions in hospital as some of them may actually have undiagnosed diabetes. Although we have included subjects aged 16 to 18 years in this study, there was only 55 of them and thus unlikely to impact the results. Furthermore, patients in this age range are admitted and managed by the adult services as a policy in this hospital.

We did not account for multiple testing in the reported analyses, but significant reductions persist for hypoglycemia rates, SD of glucose values, and recurrent hypoglycemia rates after Bonferroni correction [38]. For the analysis of effect modification by type of ward, we had classified wards as medical or surgical based on the predominant type of patients they admitted. However, the difference between medical and surgical might be diluted due to admission of medical patients to surgical wards. Our results for the second comparison may be biased towards null because of the way that the intervention status of admissions spanned across different time periods was defined. For example, an admission started in pre-implementation period but ended in post-implementation period 1 would be affected during the post-implementation period if there was an effect. However, by classifying it as a pre-implementation admission, the effect of implementation on this admission was diluted. As this was a retrospective cohort study, we could not exclude other temporal effects that could have resulted in improvement in glucometrics. We mitigated this by including characteristics of the admissions into the regression model, which may capture the temporal effects to some extent. We were not able to compare the intervention group with a control group from another hospital in which interventions were not implemented during the same time period. Future studies in other hospitals may consider such study designs to improve analysis of their program effectiveness. We also do not have longer-term data to assess the sustainability of the program beyond 8 months after completion, which will be the subject of future work.

## Conclusions

In conclusion, we have described a pragmatic multi-faceted strategy to improve inpatient diabetes care in non-critical wards. We were able to achieve sustained improvements in clinically relevant outcomes. Our results also suggest that the program was more effective in reducing hyperglycemia in medical wards. More targeted approaches may be needed to address hyperglycemia in surgical wards. Our described intervention could be feasibly implemented by other secondary and tertiary care hospitals.

## Supplementary information

**Additional file 1: Table S1.** Profiles of admissions within the 3 months period prior implementation, the first 3 months and subsequent 3 months of post-implementation at each of the 7 phases. **Table S2.** Comparison of key patient-day and admission glucometrics within the 3 months period prior implementation, the first 3 months and subsequent 3 months of post-implementation at each of the 7 phases

## Data Availability

The datasets used and/or analysed during the current study are available from the corresponding author on reasonable request.

## Abbreviations

BG: Blood Glucose
CI: Confidence Interval
DM: Diabetes Mellitus
GEE: Generalised Estimating Equations
GEEPACK: Generalised Estimating Equation Package
HGI: Hyperglycemia Index
IT: Information Technology
LOS: Length Of Stay
OR: Odds Ratio
POC: Point-Of-Care
SD: Standard Deviation
