# Supplementary material for: Evaluating the effectiveness of a multi-faceted inpatient diabetes management program among hospitalised patients with diabetes mellitus
Source: Clin Diabetes Endocrinol. 2020 Nov 5;6:21. doi: 10.1186/s40842-020-00107-2 (PMC7643419; doi:10.1186/s40842-020-00107-2)
Supplement: Supplementary file 1 — Additional file 1: Table S1. Profiles of admissions within the 3 months period prior implementation, the first 3 months and subsequent 3 months of post-implementation at each of the 7 phases. Table S2. Comparison of key patient-day and admission glucometrics within the 3 months period prior implementation, the first 3 months and subsequent 3 months of post-implementation at each of the 7 phases [file 40842_2020_107_MOESM1_ESM.docx]

**SUPPLEMENTARY MATERIAL**

**Table S1: Profiles of admissions within the 3 months period prior implementation, the first 3 months and subsequent 3 months of post-implementation at each of the 7 phases**

|  | 3 months Pre-implementation | 1^st^ to 3^rd^ months Post-implementation | | 4^th^ to 6^th^ months Post-implementation | Overall  p value † |
| --- | --- | --- | --- | --- | --- |
| Admissions | 2802 | 2879 | | 2531 |  |
| DEMOGRAPHIC | | | | | |
| Age, mean (SD) | 66.7 (14.0) | 66.2 (13.8) | 66.1 (13.6) | | 0.204 |
| Male, n (%) | 1444 (51.5) | 1557 (54.1) | 1415 (55.9) | | 0.006 |
| Ethnicity, n (%) |  |  |  | |  |
| Chinese | 1646 (58.7) | 1639 (57.9) | 1466 (57.9) | | 0.91 |
| Indian | 353 (12.6) | 387 (13.4) | 349 (13.8) | |  |
| Malay | 555 (19.8) | 601 (20.9) | 504 (19.9) | |  |
| Others | 248 (8.9) | 252 (8.8) | 212 (8.4) | |  |
| ADMISSION CHARACTERISTICS | | | | | |
| Admissions to, n (%) |  |  |  | |  |
| Medical wards | 2246 (80.2) | 2288 (79.5) | 2034 (80.4) | | 0.933 |
| Surgical wards | 498 (17.8) | 532 (18.5) | 445 (17.6) | |  |
| Others ‡ | 58 (2.1) | 59 (2.1) | 52 (2.1) | |  |
| Admission via emergency, n (%) | 1881 (67.1) | 1968 (68.4) | | 1614 (63.8) | 0.001 |
| With severe illness, n (%) § | 2514 (89.7) | 2527 (87.8) | | 2195 (86.7) | 0.003 |
| INFORMATION USED TO IDENTIFY ADMISSIONS WITH DM | | | | | |
| DM medication was prescribed, n (%) | 2467 (88.0) | 2559 (88.9) | | 2257 (89.2) | 0.394 |
| Any capillary blood glucose reading > 11.1 mmol/L, n (%) | 644 (23.0) | 677 (23.5) | | 595 (23.5) | 0.866 |
| Any HbA1c > 6.5% or 48 mmol/L, n (%) | 2398 (85.6) | 2445 (84.9) | | 2117 (83.6) | 0.137 |

†For age, F-test was performed to obtain p value. For ethnicity and type of wards, chi-square test was performed to obtain p value. For the rest of the variables, Fisher’s exact test was performed to obtain p values.

‡Others include patients who were transferred across types of wards during admission, or admitted to a non-medical and non-surgical ward.

§An admission was considered as having severe illness if the patient had any of the following lab tests records during the entire hospital stay, albumin, creatinine, C - reactive protein, white blood cells, and troponin I.

**Table S2: Comparison of key patient-day and admission glucometrics within the 3 months period prior implementation, the first 3 months and subsequent 3 months of post-implementation at each of the 7 phases**

|  | **3 months Pre-implementation** | **1^st^ to 3^rd^ months Post-implementation** | **4^th^ to 6^th^ months Post-implementation** | **Effect measures †**  **(95% CI)**  **(1^st^ to 3^rd^ months Post-implementation vs**  **3 months Pre-implementation)** | **Effect measures †**  **(95% CI)**  **(4^th^ to 6^th^ months Post-implementation vs**  **3 months**  **Pre-implementation)** | **Overall**  **p value #** |
| --- | --- | --- | --- | --- | --- | --- |
| **Patient-days** | 17316 | 18897 | 16374 |  |  |  |
| **Admissions** | 2802 | 2879 | 2531 |  |  |  |
| **HYPOGLYCAEMIA METRICS** | | | | | | |
| **Patient-day level** | | | | | | |
| **Hypoglycaemia, any glucose, n (%)** | | | | | | |
| < 4 mmol/L | 714 (4.1) | 695 (3.7) | 523 (3.2) | 0.89 (0.77 to 1.04) | 0.78 (0.66 to 0.91) | 0.006 |
| < 3 mmol/L | 101 (0.6) | 94 (0.5) | 72 (0.4) | 0.86 (0.64 to 1.17) | 0.75 (0.53 to 1.07) | 0.264 |
| < 2.5 mmol/L ‡ | 33 (0.2) | 25 (0.1) | 27 (0.2) | 0.73 (0.41 to 1.29) | 0.85 (0.47 to 1.54) | 0.558 |
| **Admission level** | | | | | | |
| **Hypoglycaemia, any glucose, n (%)** | | | | | | |
| < 4 mmol/L | 444 (15.8) | 411 (14.3) | 324 (12.8) | 0.89 (0.77 to 1.03) | 0.81 (0.69 to 0.94) | 0.023 |
| < 3 mmol/L | 91 (3.2) | 86 (3) | 53 (2.1) | 0.92 (0.68 to 1.24) | 0.66 (0.47 to 0.94) | 0.063 |
| < 2.5 mmol/L ‡ | 32 (1.1) | 22 (0.8) | 20 (0.8) | 0.71 (0.42 to 1.21) | 0.7 (0.39 to 1.26) | 0.345 |
| **Recurrence of hypoglycaemia, number of days with any glucose < 4 mmol/L, n (%)** | | | | | | |
| Exactly 1 day § | 269 (9.6) | 259 (9) | 238 (9.4) | 0.93 (0.78 to 1.11) | 0.97 (0.8 to 1.16) | 0.730 |
| 2 or more days § | 175 (6.2) | 152 (5.3) | 86 (3.4) | 0.82 (0.65 to 1.03) | 0.54 (0.41 to 0.71) | < 0.001 |
| **HYPERGLYCAEMIA AND OTHER METRICS** | | | | | | |
| **Patient-day level** | | | | | | |
| **Hyperglycaemia, any glucose, n (%)** | | | | | | |
| > 14 mmol/L | 5100 (29.5) | 5186 (27.4) | 4134 (25.2) | 0.93 (0.81 to 1.08) | 0.86 (0.75 to 0.99) | 0.110 |
| > 20 mmol/L | 968 (5.6) | 985 (5.2) | 716 (4.4) | 0.95 (0.81 to 1.11) | 0.79 (0.68 to 0.93) | 0.013 |
| **Mean glucose in desired range, n (%)** | | | | | | |
| Within 4-10 mmol/L | 10362 (59.8) | 11692 (61.9) | 10413 (63.6) | 1.04 (0.9 to 1.19) | 1.11 (0.96 to 1.29) | 0.321 |
| **Mean glucose, mean (SD)** | 9.8 (3.4) | 9.7 (3.2) | 9.5 (3.1) | 1 (0.97 to 1.03) | 0.98 (0.95 to 1.01) | 0.422 |
| **SD of glucose, mean (SD)** | 2.3 (1.6) | 2.3 (1.6) | 2.3 (1.6) | 1 (0.96 to 1.03) | 0.97 (0.93 to 1) | 0.167 |
| **Admission level** | | | | | | |
| **Hyperglycaemia, any glucose, n (%)** | | | | | | |
| > 14 mmol/L | 1578 (56.3) | 1560 (54.2) | 1328 (52.5) | 0.94 (0.85 to 1.04) | 0.89 (0.8 to 0.99) | 0.117 |
| > 20 mmol/L | 526 (18.8) | 483 (16.8) | 391 (15.4) | 0.87 (0.75 to 1.01) | 0.82 (0.71 to 0.96) | 0.035 |
| **Mean glucose in desired range, n (%)** | | | | | | |
| Within 4-10 mmol/L | 1589 (56.7) | 1733 (60.2) | 1516 (59.9) | 1.14 (1.02 to 1.27) | 1.09 (0.97 to 1.21) | 0.051 |
| **HGI, mean (SD)** | 0.49 (0.95) | 0.47 (1.01) | 0.42 (0.9) | 0.84 (0.66 to 1.07) | 0.77 (0.6 to 0.98) | 0.102 |
| **Mean glucose, mean (SD)** | 9.9 (2.7) | 9.8 (2.7) | 9.7 (2.6) | 0.99 (0.98 to 1.01) | 0.99 (0.98 to 1.01) | 0.455 |
| **SD of glucose, mean (SD)** | 2.85 (1.4) | 2.81 (1.4) | 2.75 (1.4) | 0.99 (0.96 to 1.01) | 0.97 (0.94 to 1) | 0.092 |
| **LOS (in days), mean (SD) ¶** | 8.5 (12) | 8.0 (10.4) | 6.8 (6.6) | 0.96 (0.9 to 1.02) | 0.85 (0.8 to 0.9) | < 0.001 |

†The associations between glucometrics and the time periods were fully adjusted with respect to age, gender, ethnicity groups, emergency admission, type of ward, and illness severity status. For mean glucose, SD (standard deviation) of glucose, HGI (Hyperglycaemia Index) with the threshold being 14 mmol/L, and LOS (length of stay), Poisson regression model with Generalized Estimation Equations (GEE) was applied and mean ratios were reported with 95% CI within the brackets. For other metrics, logistic regression model with GEE was applied and odds ratios were reported with 95% CI within the brackets.

‡Blood glucose < 2.5 mmol/L is considered biochemically severe hypoglycaemia.

§For recurrence of hypoglycaemia, the comparison reference group are always admissions without any glucose < 4 mmol/L, i.e., zero days with any glucose < 4 mmol/L.

**#**Overall p value was reported to test the overall significant difference for the glucometrics across the three time periods.

¶The associations between length of stay and the time periods were fully adjusted with respect to age, gender, ethnicity groups, emergency admission, type of ward, illness severity status, and glucometrics.
